# Efficacy and safety of programmable compared with fixed anti-siphon devices for treating idiopathic normal-pressure hydrocephalus (iNPH) in adults – SYGRAVA: study protocol for a randomized trial

**DOI:** 10.1186/s13063-018-2951-6

**Published:** 2018-10-17

**Authors:** Romy Scholz, Johannes Lemcke, Ullrich Meier, Dirk Stengel

**Affiliations:** 1 Centre for Clinical Research, Unfallkrankenhaus Berlin, Warener Str. 7, 12683 Berlin, Germany; 2 Department of Neurosurgery, Unfallkrankenhaus Berlin, Warener Str. 7, 12683 Berlin, Germany; 3 Department of Trauma and Orthopaedic Surgery, Unfallkrankenhaus Berlin, Warener Str. 7, 12683 Berlin, Germany; 4 Julius Wolff Institute, Charité Medical University Centre, Augustenburger Platz 1, 13353 Berlin, Germany

**Keywords:** Idiopathic normal-pressure hydrocephalus, Randomized trial, Dementia, Ventriculoperitoneal shunt, Programmable anti-siphon device

## Abstract

**Background:**

Idiopathic normal-pressure hydrocephalus (iNPH) is a distinct form of dementia, characterized by gait ataxia, cognitive impairment and urinary incontinence. In contrast to all other causes of dementia (e.g., Alzheimer-type and others), ventriculoperitoneal (VP) shunt surgery may offer a curative treatment option to patients. While being a rather low-risk type of surgery, it may cause significant over- or underdrainage complications (e.g., headaches, dizziness, vomiting, intracerebral bleeding, etc.) during posture change. Anti-siphon devices (ASDs) are a group of technically different additional valves used in shunt surgery. They are designed to maintain intraventricular pressure within a normal physiological range regardless of patient position. Fixed ASDs proved to substantially lower the rate of overdrainage complications. No significant differences, however, were noted regarding underdrainage complications. Technical successors of fixed ASDs are programmable ASDs. The aim of this study is to evaluate whether programmable ASDs compared to fixed ASDs are able to avoid both over- and underdrainage complications.

**Methods/design:**

In this investigator-initiated, multicenter randomized trial, 306 patients are planned to be recruited. Male and female patients aged ≥18 years with iNPH who are eligible for VP shunt surgery and meet all other entry criteria can participate. Patients will be randomized in a balanced 1: 1 fashion to a VP shunt with a programmable valve either supplemented with a fixed ASD, or a programmable ASD. Patients will be followed-up 3, 6 and, on an optional basis, 12 months after surgery. The primary outcome measure is the cumulative incidence of over- or underdrainage 6 months post surgery, as defined by clinical and imaging parameters.

**Discussion:**

SYGRAVA is the first randomized trial to determine whether programmable ASDs reduce complications of drainage compared to fixed ASDs in patients with iNPH. The results of this study may contribute to health-technology assessment of different valve systems used for VP-shunt surgery, and determination of the future standard of care.

**Trial registration:**

International Standard Randomised Controlled Trial Number: ISRCTN13838310. Registered on 10 November 2016.

**Electronic supplementary material:**

The online version of this article (10.1186/s13063-018-2951-6) contains supplementary material, which is available to authorized users.

## Background

Idiopathic normal-pressure hydrocephalus (iNPH) is a syndrome characterized by ataxia, cognitive impairment and urinary incontinence (Hakim’s triad). Neuro-imaging frequently shows dilated ventricles along with normal cerebrospinal fluid (CSF) pressure at lumbar puncture [1, 2]. Modern pathophysiological understanding assigns iNPH to sclerosis of the cerebral arteries which impairs the ability of modulating pressure peaks, increases trans-cerebral mantle pressure gradients and subsequent leads to ventricular dilatation [3].

Most resilient epidemiological data on iNPH come from Scandinavia. Eide and colleagues found an annual incidence rate of 1.09 / 100,000 in the general population of a southern Norwegian province. In subjects older than 65 years, the incidence rate was 30.2 / 100,000 [4]. By computed tomography (CT) screening of 1238 patients, Wikkelsø et al. determined a prevalence of iNPH of 0.2 and 5.9% among 70- to 79- and ≥ 80-year-old subjects, respectively [5].

INPH can surgically be treated by diversion of the CSF [6]. Implanting a shunt to drain the CSF to the peritoneal (ventriculoperitoneal, VP) or pleural cavity, or the venous system (ventriculoatrial, VA), may significantly and immediately improve symptoms and gait [7]. VP shunting using programmable valves is probably the most widely used surgical option for managing patients with iNPH. Yet, shunting harbors the risk of complications resulting from overdrainage such as hygroma or subdural bleeding. This may cause severe headache and nausea, and subsequently demand revision surgery. Hydrostatic pressure changes in a VP shunt are posture dependent. Valves programmed to provide adequate intraventricular and shunt pressure in the horizontal position may rapidly change to overdrainage in the upright position. If the valve pressure is set too low in the upright position, underdrainage may occur in the horizontal position, compromising the benefits of shunt surgery.

**Fig. 1:**
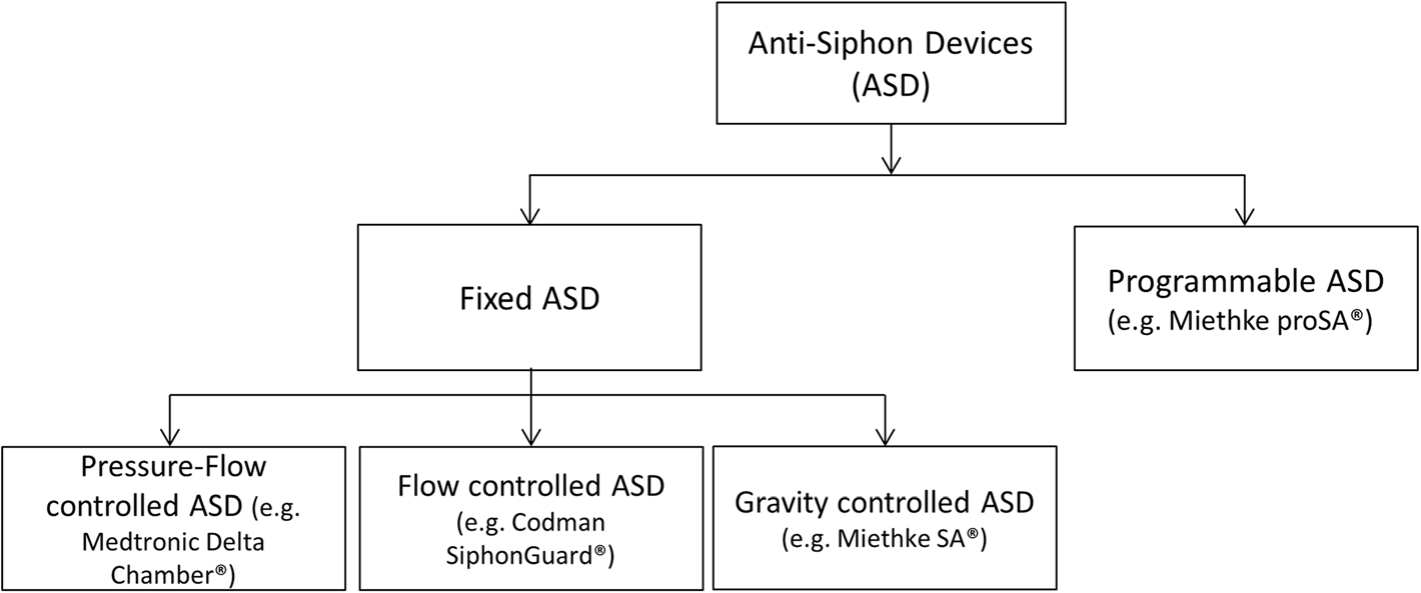
Types of anti-siphon devices (ASDs) – programmable ASD and fixed ASD (pressure-flow-controlled, flow-controlled and gravity-controlled)

Anti-siphon devices (ASDs) are a group of technically different additional valves designed to reduce the potential hazards of excessive lowering of intraventricular pressure (Fig. 1). They keep intraventricular pressure in a normal physiological range, regardless of the patient’s position.

The following systems are established, approved and commercially marketed in both the US and Europe:

### Fixed ASDs

- Delta Chamber® (Medtronic Inc.): the Delta Chamber® is part of the STRATA II® valve. It is a membrane valve designed to minimize the CSF flow in a siphoning situation in order to prevent overdrainage. The Delta Chamber® is also available as an individual part and can be connected to other devices in a serial fashion
- SiphonGuard® (Codman, Johnson & Johnson): the Codman SiphonGuard® is an optional component of the Codman® HAKIM® Programmable Valve and the Codman® CERTAS® / CERTAS® Plus Programmable Valve. Its anti-siphon effect is based on a secondary CSF pathway with a significantly reduced flow rate inside the valve activated by a switch in the form of a ball and spring valve. The SiphonGuard® is also available as an individual part and can be serially connected to other valves
- ShuntAssistant® (Miethke, Germany): the ShuntAssistant® is a detachable part of the Miethke proGAV® valve. The gravity of a tantalum ball is the counterweight to negative hydrostatic pressure in the shunt system. The ShuntAssistant® is designed to compensate the additional hydrostatic pressure difference within the shunt system in the upright position. The ShuntAssistant® is also available as an individual part

### Programmable ASDs

- proSA® (programmable ShuntAssistant) (Miethke, Germany): currently, the proSA® is the only available programmable ASD and can provide different independent pressure settings for the upright position. It is available as part of assembled valve combinations and as a single part. The underlying technical principle is the gravity-dependent regulation of the opening pressure by a tantalum weight, which is mounted on a lever arm with adjustable pre-load

According to bench tests [8], all mentioned devices work as intended but differ in their mechanism of action. For the group of gravitational valves, clinical trials suggest effectiveness in avoiding drainage complications [9, 10]. Other ASDs were studied only under laboratory conditions or in the context of secondary analyses in clinical investigations [8, 11].

We hypothesize that programmable ASDs compared to fixed ASDs reduce the risk of both, over- and underdrainage.

## Methods/design

### Design overview

**Fig. 2:**
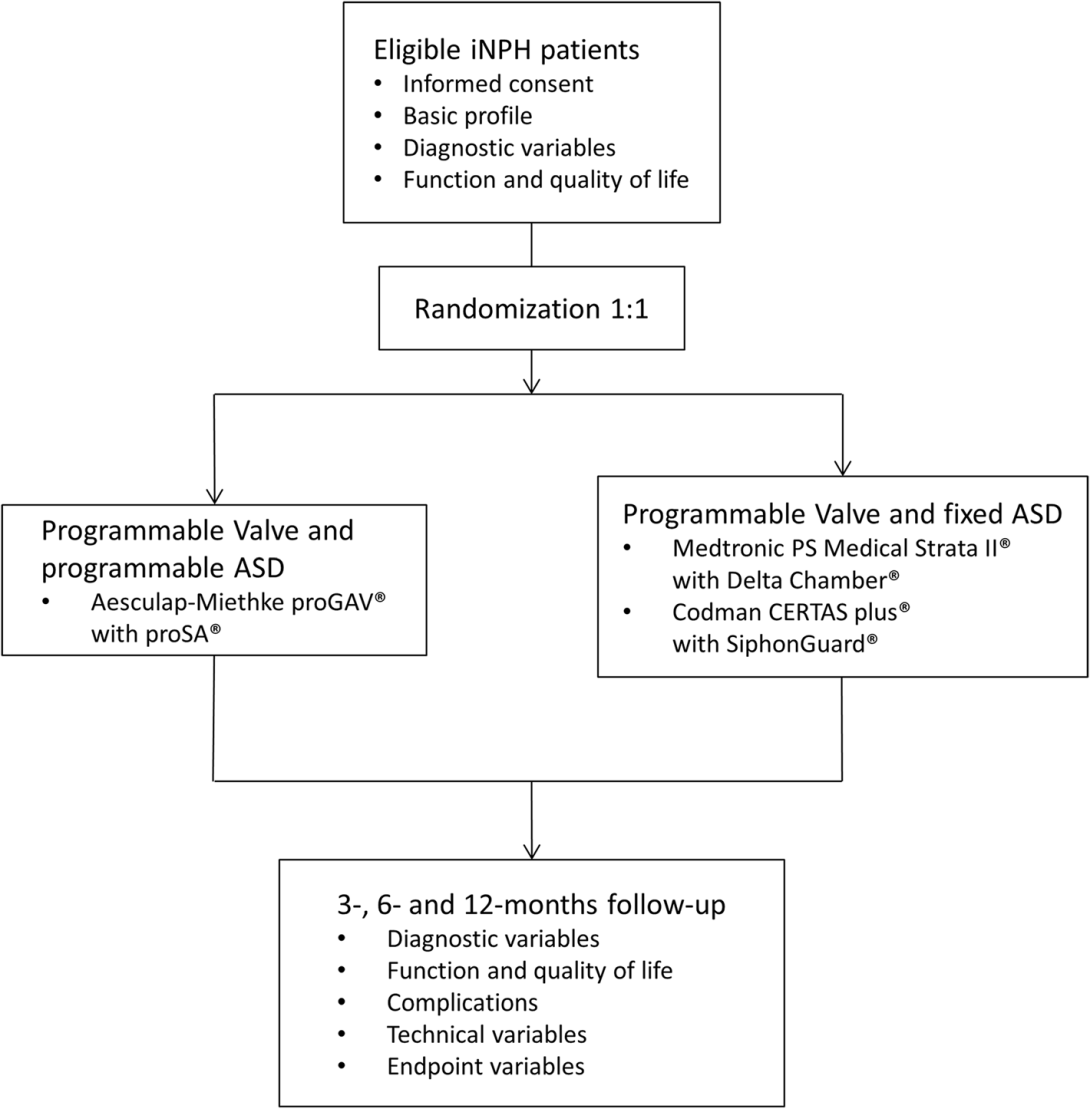
Trial flow diagram

This is a pragmatic, open-label, multicenter randomized trial based on an alpha-adaptive design and a 1: 1 allocation ratio (Fig. 2). The study compares the rate of all drainage complications between fixed and programmable ASDs. The trial will be conducted at neurosurgical units with renowned expertise and high surgical volumes. Diagnosis of iNPH will be made in accordance with locally established algorithms and guideline-compliant tests in each center. Single non-invasive, invasive, and a combination of tests and imaging findings can be used to confirm the diagnosis of iNPH.

Patients will randomly be allocated to receive a VP shunt with a programmable valve and programmable ASD or a VP shunt with a programmable valve and a fixed ASD. Follow-up visits will be performed 3, 6, and, on an optional basis, 12 months after surgery.

After start of recruitment and first patient in, the study is expected to run for 4 years (3 years of recruitment plus a maximum of 12 months of follow-up).

### Diagnostic criteria for confirming the presence of iNPH

#### Clinical

Tentative clinical diagnosis of iNPH is based on thorough neurological examination and the occurrence of the Hakim’s triad. Gait disturbance is pathognomonic and typically appears as a slow, shuffling, small-step, broad-based and unsafe movement pattern [12, 13]. According to the literature, 86 to 100% of all iNPH patients show ataxia [14–16], which is a requirement for iNPH diagnosis in the present study.

Cognitive impairment can be pronounced (short-term memory disorders) or even absent. There is significant variation in the severity of symptoms [12, 14, 17–19]. Psychopathological signs may range from apathy to indifference or even akinetic mutism. Dyscalculia and acalculia [20], disorientation [21], depression and anxiety [22] may be detectable as well.

In the early stages of iNPH, incontinence manifests as urinary urgency or urge incontinence. This may progress to a complete loss of control of bladder function. Clinical signs and symptoms will be documented using the Kiefer score [23].

#### Technical, invasive / non-invasive

Enlarged ventricle width on CT or magnetic resonance imaging (MRI) scans is required for confirming the diagnosis of iNPH. The Evans index (the ratio of maximum width of the frontal horns of the lateral ventricles and maximal internal diameter of skull at the same level) is an accepted morphological indicator of ventricular enlargement and must be ≥ 0.3.

Cerebrospinal fluid flow rates in functional MRI may be sufficiently accurate to diagnose iNPH [24]. For the quantitative 2-D phase-contrast technique, a flow rate of > 24.5 ml / min is 95% specific in proving the presence of iNPH. However, this non-invasive method is less sensitive (46%) and must not be used to exclude iNPH.

Long-term measurement of ICP signals over at least 24 to 72 h might be performed. B-waves of the ramp type and an increased B-wave activity may be regarded as a pathognomonic sign of iNPH. Likewise, increased ICP pulse amplitudes (in terms of quantification of pulsatility (Q-pulse) [25]) will be considered as predictive variables. A non-invasive measurement using solely otoacoustic emissions is not sufficient.

Both static and dynamic protocols of the lumbar infusion test may be used. According to guidelines for management of iNPH of the Japanese Neurosurgical Society [26] and the guidelines of the American iNPH Study Group [27], the positive predictive value (PPV) of cerebrospinal outflow resistance (infusion test) ranges between 75 and 92%. The sensitivity and specificity of the infusion test are estimated at 58 to 100% and 44 to 92%, respectively.

A ventricular infusion test is performed only in patients with severe degenerative changes of the lumbar spine which prohibit uncomplicated lumbar puncture.

A cerebrospinal tap test can either be carried out in combination with a lumbar infusion test or separately. Kahlon et al. state the positive predictive value of the cerebrospinal tap test to be 94% [28].

Clinical control of gait disturbance should occur 2 to 4, 24, 48 and 72 h after spinal tap according to a fixed scheme. We recommend the 10-m walk test and the 360° test, each with documentation of the number of steps and time in seconds.

An external lumbar drainage can be used as the initial diagnostic tool or as a back-up option in case of equivocal or unclear results from previous examinations. The lumbar drainage should be maintained for a period of 72 h [29].

#### Hypothesis

In patients undergoing VP-shunt surgery for iNPH, the use of programmable valves with programmable ASDs lowers the composite incidence of under- and overdrainage complications from 27 to 10% at 6 months after surgery compared to the standard of care (i.e., programmable valves with fixed ASDs). This translates to a risk difference (RD) of 17%, a risk ratio (RR) of 0.37 and a relative risk reduction of 63%.

### Study population

#### Inclusion criteria

- Age ≥ 18 years
- Meet clinical, physiological, functional and radiological diagnostic criteria of iNPH
- Scheduled for VP shunting
- Capable of understanding the trial concept and its implications, and of providing written (or witnessed verbal) informed consent

#### Exclusion criteria

- Secondary NPH after infection, trauma, tumors, etc.
- Contraindication for shunt surgery (e.g., malignant disease with reduced life expectancy, florid infections, etc.)
- Advanced dementia
- Guardianship
- Any disability prohibiting informed consent
- Patients with previous shunt implantation
- Patients with previous ventriculostomy

### Randomization

Patients will randomly be assigned to the individual treatment group using a web-based randomization and documentation platform (SecuTrial™). A block randomization scheme and an allocation ratio of 1: 1 will be employed.

### Interventions

Subjects in the programmable ASD (or experimental) group will undergo VP-shunt surgery with a combination of a programmable valve and a programmable ASD (Miethke proGAV® plus proSA®). The initial setting of the proGAV® will be 70 mmH_2_O. The initial setting of the proSA® device will be determined by the surgeon. The recommended setting for the study is 200 mmH_2_O for patients shorter than 160 cm, 250 mmH_2_O for patients with a body height of 160 to 180 cm and 300 mmH_2_O for patients taller than 180 cm.

**Fig. 3:**
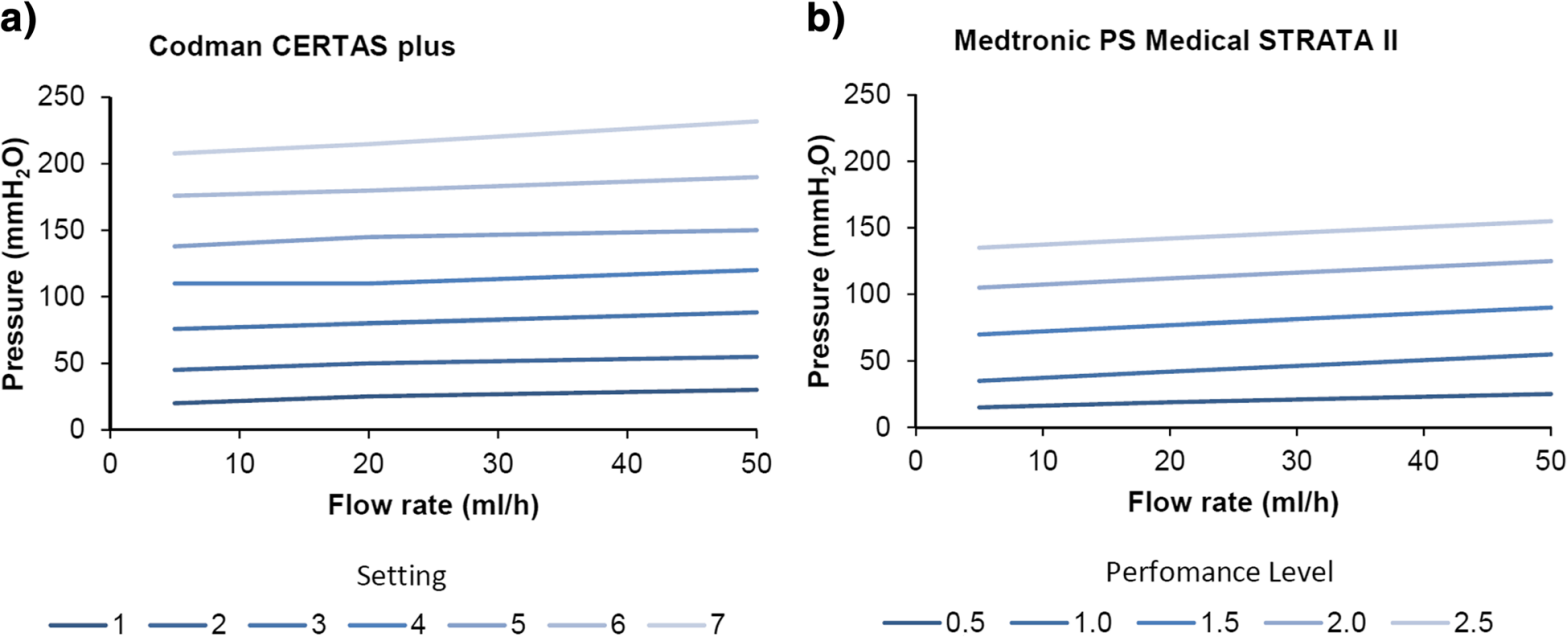
Pressure-flow curves of the adjustment settings of the Codman CERTAS plus valve® (**a**) and of the Medtronic PS Medical STRATA II® valve (**b**) modified according to (**a**) Codman CERTAS plus brochure DSUS/COD/1214/0229a 01/15 and (**b**) Medtronic PS Med STRATA II instructions for use 16,417-1H 30,329

Patients in the fixed ASD (or control) group will receive a VP shunt with a programmable valve plus a fixed ASD (Medtronic PS Medical Strata II® valve with Delta Chamber® or Codman CERTAS plus® with SiphonGuard® depending on the preference or availability at individual centers). To ensure a similar function of the low pressure units in both arms, the valves in this group will be set as shown in Table 1 based on the corresponding pressure-flow curves and depending on the setting given by the manufacturers (Fig. 3).

**Table 1:** Initial setting of valves in the control group

Valve type	Setting	mmH_2_O
PS Medical Strata II valve®	Performance level 1.5	70
Codman CERTAS plus Programmable Valve®	Setting 3	80

**Fig. 4:**
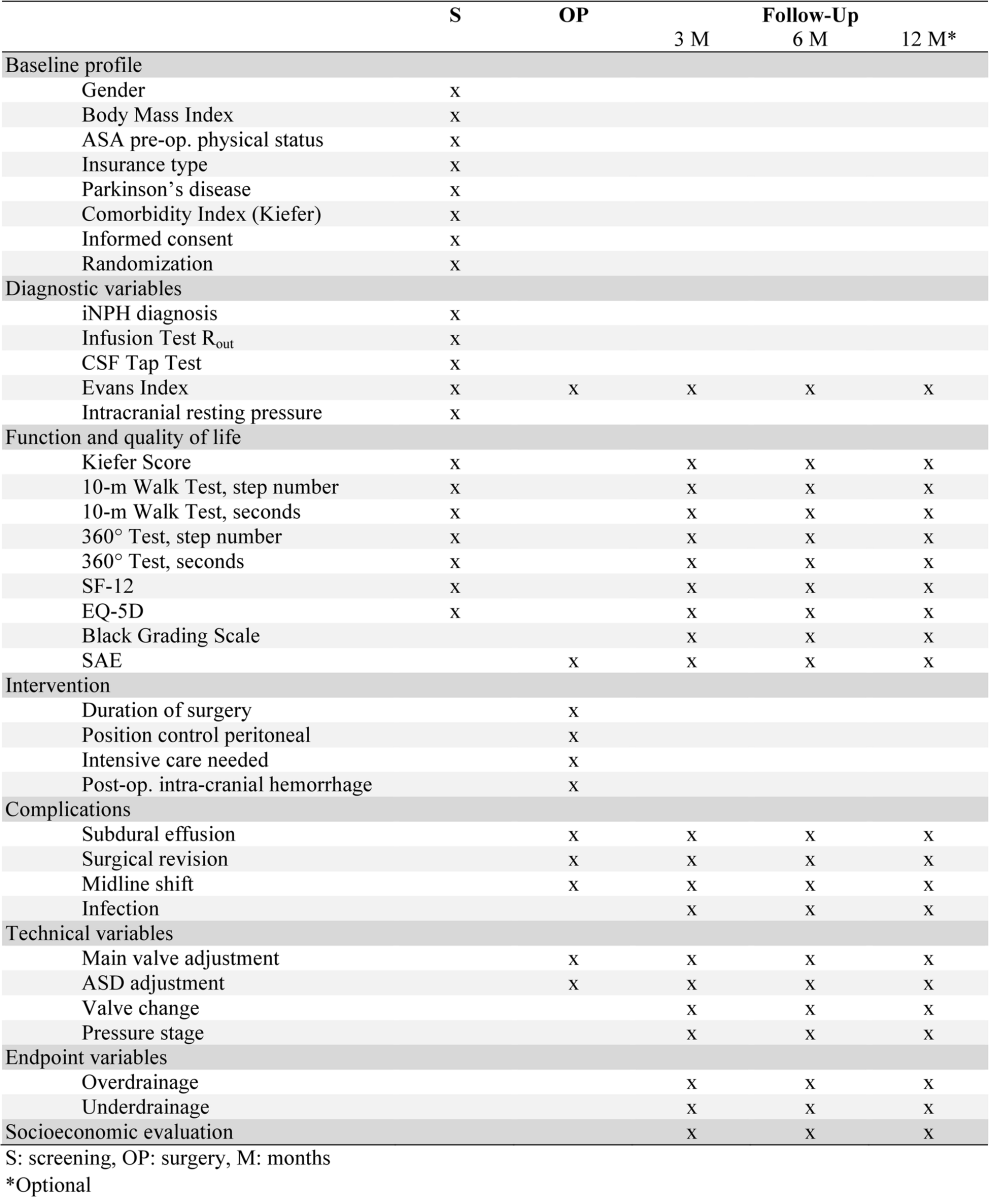
Schedule of enrollment, interventions and assessments

Documentation will be performed at five designated visits (Fig. 4). First is screening, second is surgery, with further follow-up visits scheduled at 3 months (± 1 week) and 6 months (± 2 weeks) after surgery. We also plan a long-term follow-up after 12 months (± 4 weeks).

### Statistical planning and sample size calculation

Available data on the risk of drainage-associated complications vary among different types of ASD. Publications including the cumulative rate of drainage complications of flow-controlled and pressure-flow-controlled conventional ASDs do not exist.

The rate of overdrainage of the pressure-flow-controlled Codman SiphonGuard® subgroup in a non-randomized study was 16% [11]. Rates of underdrainage have not been published.

The overdrainage rate in a randomized trial enrolling patients who were treated with the Medtronic Delta Chamber® (flow-controlled fixed ASD) as part of the STRATA II valve was 5 and 28%, respectively, depending on the selected performance level of the valve [30]. Given that only the lower of the two pressure settings for the main valve is effective for iNPH treatment, the cumulative rate of overdrainage is estimated to be 28%.The composite rate of over- and underdrainage complications 6 months after implantation of non-programmable gravitational units (gravity-controlled fixed ASD) can be estimated at 10% (7 out of 74 patients) based on preliminary work by our group [9, 10].

Taking all previous investigations and clinical observations together, we presume a 27% incidence of drainage complications in the control group, provided that the two fixed ASDs are used to the same extent during the study. As the experimental device is technically based on the gravity-controlled fixed ASD, a maximum 10% cumulative rate of drainage complications is assumed for the experimental arm.

In the light of the available evidence, the following clinically important and methodologically sound assumptions were made:

1. The occurrence of drainage complications of any kind, although a surrogate, is relevant to patients, payers and health-care services and systems, and was consented as the primary endpoint of the trial by both the principal and all other investigators, and members of the Trial Steering Committee
2. The combined rate of over- and underdrainage complications after implantation of a fixed ASD is 27%
3. Through a programmable unit this rate will be lowered to 10%
4. Due to the anticipated high prevalence of comorbidities the lost-to-follow-up and drop-out rate is 30%
5. The study should be able to detect existing treatment effects with a probability (power) of 85% (including a power reserve)
6. A conservative two-sided type I error of 5% is accepted

To prove a risk difference (RD) of 17% under the assumptions mentioned above, 2 × 107 evaluable patients will be needed in the modified intent-to-treat (mITT) population.

We will exclude patients who die in the first short interval between randomization and surgery, who withdraw their consent for study participation, are acutely not suitable for surgery, or who sustain an emergent medical event which prevents shunt surgery.

A conservative ITT analysis will be carried out additionally. This will be supplemented by per-protocol- and as-treated analyses. To compensate for drop-outs and a lost-to-follow-up rate of 30%, a total of 2 × 153 = 306 patients will be recruited onto the trial.

Accounting for test results under laboratory conditions, there are no reasons for assuming different hydrodynamic properties of the programmable main valves concerning their hydrodynamic resistance, their flow-pressure dependency and their constancy of opening and closing pressures under physiological conditions [31–33].

### Composite endpoint: over- and underdrainage

The primary outcome of this trial is the cumulative incidence of over- or underdrainage at 6 months post surgery. This is a binary composite outcome measure based on the following individual signs and symptoms or their combination:

- Subdural effusions ≥ 3 mm
- Subdural effusion mandating surgical intervention
- Clinical symptoms of overdrainage (e.g., headache, dizziness, nausea, vomiting) in combination with a decrease in Evans index of at least 0.03
- Slit-ventricle syndrome
- Increase in disease-specific clinical symptoms (Kiefer score ≥ 2 points) in combination with an increase in the Evans index of at least 0.03 while proving permeability of the shunt
- Symptomatic obstruction of an ASD
- Conversion of the opening pressure of the proGAV® adjustment unit to ≥ 90 mmH_2_O
- Revision of the proGAV® adjustment unit to a medium- or high-pressure valve or a programmable valve set to medium- or high-pressure range (≥ 90 mmH_2_O or performance level ≥ 2.0)
- Indication for an additional endoscopic third ventriculostomy
- Ligation of a shunt
- Implantation of additional ASDs
- Replacement of the ASD

### Secondary outcomes

- Slit-ventricle syndrome as detected by CT imaging after 3, 6 and 12 months
- Subdural effusions as detected by CT imaging after 3, 6 and 12 months
- Infections measured by clinical evaluation after 3, 6 and 12 months
- Neuro-functional outcomes measured by Kiefer score, Black Grading Scale, 10 min walk test and 360° test after 3, 6 and 12 months

Health-related quality of life measured by the Short-Form 12 (SF-12) and the EuroQol five-dimensions quality of life (EQ-5D) score after 3, 6 and 12 months

### Early termination

An adaptive O’Brien-Fleming design with one planned interim analysis halfway will assist researchers and sponsors to decide about necessary protocol adaptations or even early termination (e.g., in case of a clear advantage of the experimental treatment, a *p* value < 0.003 for primary endpoint assessment using an unadjusted z-test, futility, compromised patient safety, etc.).

### Statistical analysis

All biostatistical methods are in line with international recommendations and the current state of medical research. Depending on the quality and structure of data, absolute values, proportions, means and medians with adequate indicators and measures of distribution (standard deviations, interquartile ranges (IQRs, ranges, 95% confidence intervals (CI)) will be reported. The primary endpoint analysis will be performed using logistic regression. Secondary endpoint and further analyses are based on the type of outcome and exposure variables.

All analytical procedures will be described in a separate Statistical Analysis Plan (SAP) supplying the Clinical Investigation Plan (CIP). Any necessary deviations from the original analytical concept conditional on the observations will be explained.

### Trial registration and dissemination

This study was registered with ISRCTN (ISRCTN13838310). A Standard Protocol Items: Recommendations for Interventional Trials (SPIRIT) Checklist is provided as Additional file 1. The findings of this trial will be submitted to a peer-reviewed journal and abstracts will be presented at relevant national and international conferences.

### Trial participating centers

Departments of Neurosurgery at the Unfallkrankenhaus Berlin, Dietrich-Bonhoeffer-Hospital Neubrandenburg, Saarland University Medical Center Homburg / Saar, University Medical Centre Göttingen, Heidelberg University Hospital and University Medicine Greifswald, Germany.

## Discussion

More than 100 different valve types for the treatment of iNPH achieved market approval and are used in clinical practice [34]. So far, less than ten randomized controlled trials on this particular subject were conducted, and only few valves have been systematically evaluated.

Nevertheless, several trials dramatically changed the standard of care for iNPH. The Dutch NPH study [35] established the concept of low valve-opening pressures, Zemack and Romner [36] launched programmable shunt valves as a standard of care, and the SVASONA trial [10] demonstrated the effectiveness of non-programmable ASDs in addition to programmable shunt valves. Overdrainage complications are almost completely avoidable with these devices while underdrainage remains a problem.

Evidence for effectiveness of programmable ASDs in preventing over- and underdrainage is pending.

### Inclusion criteria

Only patients with iNPH will be included in the study. Since no “gold standard” for the diagnosis of iNPH exists, even the most experienced centers cannot provide identical diagnostic pathways. Therefore, we decided to allow for any established diagnostic approach aiming at identifying patients with iNPH at individual trial sites (given that they suit common clinical concepts and best-practice guidelines). The reason for this decision is that specificity and sensitivity of diagnostic tests for iNPH cannot be calculated without restrictions since the number of false-negative findings remains unclear. Accordingly, the best benchmark for these diagnostic tests is the responder rate after shunt surgery. Since the responder rates of ICP measurement during 24 to 72 h, B-wave analysis, ICP pulse-amplitude analysis and lumbar infusion test are comparable, these diagnostic tests are suitable for this study.

### Intervention

Recent studies showed the effectiveness of non-programmable ASDs in preventing overdrainage complications among patients with iNPH. Since underdrainage is a problem caused by excessive valve-opening pressure, which in turn is related to poorer outcomes, the individual adjustment of the ASD in the upright position seems to be the key element for avoiding both over- and underdrainage. Thus, the aim of our study is to compare programmable ASDs with non-programmable ASDs with respect to all kinds of drainage complications.

For the sake of a pragmatic trial framework, main valves and ASDs are combined in a merchantable configuration. Although a cross-manufacturer configuration of main valves and ASDs proved to be possible [37], there are several practical and legal limitations restricting the external validity and transferability of trial results.

### Endpoints

The choice of both pragmatic and relevant endpoints was a critical step in the design of the study. In non-programmable shunt valves, both over- and underdrainage complications manifest with distinct imaging signs which may prompt revision surgery.

The experienced neurosurgeon is able to detect drainage complications at a subclinical state and tries to avoid their clinical manifestation by actively regulating the valve-opening pressure. This may lead to an increase of the valve-opening pressure to the high-pressure range in order to prevent a patient from imminent overdrainage. However, the resulting opening pressure precludes the therapeutic effect of the shunt.

Hence, we decided to construct a composite endpoint that covers any over- and underdrainage scenario and, by proper intervention, prevents insufficient treatment and complications. We are aware of the methodological difficulties of this complex endpoint. Yet, we think the subtle construction is both pragmatic and honest.

### Trial status

Participant recruitment is ongoing.

## Additional file

Additional file 1: Standard Protocol Items: Recommendations for Interventional Trials (SPIRIT) 2013 Checklist: recommended items to address in a clinical trial protocol and related documents. (DOC 120 kb)

## Abbreviations

ASD: Anti-sSiphon d-Device
CSF: Cerebrospinal fluid (lLiquor cerebrospinalis)
CT: Computer tomography
ICP: Intracranial pressure
iNPH: Idiopathic normal- pressure hydrocephalus
ISRCTN: International Standard Randomised Controlled Trial Number
MRI: Magnetic rResonance iImaging
Q-pulse: Quantification of pPulsatility
SF-12: Short-Form 12 Health Survey
VP: Vventriculoperitoneal
